# Analysis of methane-producing and metabolizing archaeal and bacterial communities in sediments of the northern South China Sea and coastal Mai Po Nature Reserve revealed by PCR amplification of *mcrA* and *pmoA* genes

**DOI:** 10.3389/fmicb.2014.00789

**Published:** 2015-02-17

**Authors:** Zhichao Zhou, Jing Chen, Huiluo Cao, Ping Han, Ji-Dong Gu

**Affiliations:** ^1^Laboratory of Environmental Microbiology and Toxicology, School of Biological Sciences, The University of Hong KongHong Kong, China; ^2^Division of Life Science, Hong Kong University of Science and Technology, Hong KongChina

**Keywords:** methanotrophs, anaerobic methane oxidation, carbon cycle, *mcrA* gene, *pmoA* gene

## Abstract

Communities of methanogens, anaerobic methanotrophic archaea and aerobic methanotrophic bacteria (MOB) were compared by profiling polymerase chain reaction (PCR)-amplified products of *mcrA* and *pmoA* genes encoded by methyl-coenzyme M reductase alpha subunit and particulate methane monooxygenase alpha subunit, respectively, in sediments of northern South China Sea (nSCS) and Mai Po mangrove wetland. Community structures representing by *mcrA* gene based on 12 clone libraries from nSCS showed separate clusters indicating niche specificity, while, *Methanomicrobiales, Methanosarcinales* clades 1,2, and *Methanomassiliicoccus*-like groups of methanogens were the most abundant groups in nSCS sediment samples. Novel clusters specific to the SCS were identified and the phylogeny of *mcrA* gene-harboring archaea was updated. Quantitative polymerase chain reaction was used to detect *mcrA* gene abundance in all samples: similar abundance of *mcrA* gene in the surface layers of mangrove (3.4∼3.9 × 10^6^ copies per gram dry weight) and of intertidal mudflat (5.5∼5.8 × 10^6^ copies per gram dry weight) was observed, but higher abundance (6.9 × 10^6^ to 1.02 × 10^8^ copies per gram dry weight) was found in subsurface samples of both sediment types. Aerobic MOB were more abundant in surface layers (6.7∼11.1 × 10^5^ copies per gram dry weight) than the subsurface layers (1.2∼5.9 × 10^5^ copies per gram dry weight) based on *pmoA* gene. Mangrove surface layers harbored more abundant *pmoA* gene than intertidal mudflat, but less *pmoA* genes in the subsurface layers. Meanwhile, it is also noted that in surface layers of all samples, more *pmoA* gene copies were detected than the subsurface layers. Reedbed rhizosphere exhibited the highest gene abundance of *mcrA* gene (8.51 × 10^8^ copies per gram dry weight) and *pmoA* gene (1.56 × 10^7^ copies per gram dry weight). This study investigated the prokaryotic communities responsible for methane cycling in both marine and coastal wetland ecosystems, showing the distribution characteristics of *mcrA* gene-harboring communities in nSCS and stratification of *mcrA* and *pmoA* gene diversity and abundance in the Mai Po Nature Reserve.

## INTRODUCTION

Methane is a significant greenhouse gas drawing increasingly attention from the public recently as its contribution to global warming effect accounts for nearly 20% according to the previous research (Cicerone and Oremland, 1988) and it induces more powerful greenhouse effects than carbon dioxide (Forster et al., 2007). Studies evaluating the methane emission and consumption in anoxic environments indicate that methane generation from the decomposition of organic matter in low temperature zone account for 20% of the total global methane source (Rice, 1993). Small amount of net methane emission from oceanic anoxic environment accounted for only 2% of the total atmospheric source, suggesting that it is a dynamic balance of methane consumption mediated by anaerobic oxidation of methane (AOM) serving as a methane sink (Reeburgh, 1976). Anaerobic methanotrophic (ANME) archaea apparently mediated AOM process in the forms of tight aggregates surrounded by sulfate-reducing bacteria (SRB) responsible for methane-based sulfate reduction (Boetius et al., 2000). When sulfate is not available or limited, for example, above the sulfate-methane transition zone (SMTZ), evidence showed humic substances or oxidized manganese could also serve as alternative electron acceptors (Lovley et al., 1996; Beal et al., 2009).

In terrestrial ecosystems, natural wetland is one of the important emission sources, supplying almost 20% of the total global methane emission (Cicerone and Oremland, 1988), especially in coastal and estuarine areas due to the eutrophication effect resulting from high concentrations of organic matter. Gas bubbles can be obviously visible in these areas, indicating their appropriate environmental conditions for methane production (Fleischer et al., 2001).

Methanogenesis is an important process in the carbon cycling, serving as the final step of decomposition of organic matter under anaerobic conditions when most of the electron acceptors are limited (Thauer, 1998). Methanogens occur widely producing methane in anoxic habitats, such as wetland, wastewater treatment plants, digestive tracts of cattle and humans, and marine sediments (Liu and Whitman, 2008; Li et al., 2009). Recent studies showed that methanogenesis attributed to a large fraction of methane formed and accumulated in anoxic conditions, including petroleum reservoirs (Katz, 2011; Mbadinga et al., 2011, 2012; Wang et al., 2011, 2012a,b, 2014).

All known methanogens could be divided into three types according to substrate utilization: CO_2_ reduction, methyl-containing compounds, and acetate, but all requiring the final step of methane synthesis catalyzed by the methyl-coenzyme M reductase (MCR; Liu and Whitman, 2008). Generally, methanogens are presently divided into five orders: *Methanobacteriales*, *Methanococcales*, *Methanomicrobiales*, *Methanosarcinales*, and *Methanopyrales* according to their phylogenetic distance, morphological characteristics, phospholipid compositions, etc. (Liu and Whitman, 2008).

Recent analysis of ANME systematics is based on phylogenetics of 16S rRNA and *mcrA* (methyl-coenzyme M reductase alpha subunit) genes. Three major clusters are designated to be ANME-1, 2 and 3, in which ANME-1 has been viewed as a ubiquitous cluster because of its close association with SRB and large genetic distances from the other two groups (Orphan et al., 2001, 2002; Hallam et al., 2003). On the other hand, *mcrA* gene has been classified into ANME-a, b, c, d, e, and f phylotypes. Recently, two newly identified *mcrA* gene subgroups designated as ANME-g and ANME-h have been established, in which, group g is regarded as representatives specifically adapted to terrestrial freshwater (Takeuchi et al., 2011). Through the parallel phylogenetic comparison of 16S rRNA gene and *mcrA* gene, corresponding relationships have been established as follows: *mcrA* subgroups a-b and g-h, c-d, f to be in ANME 1, 2c, 3 subtypes, respectively, while *mcrA* subgroup e was postulated to be in ANME-2a (Hallam et al., 2003; Lösekann et al., 2007).

Recently, *mcrA* gene has been frequently used as an excellent genetic marker to trace the distribution pattern of methanogens in ecosystems, because it is relatively conservative and directly catalyzes biochemical functions (Hallam et al., 2003). Not only in detecting the existence and abundance of methanogens in natural environments could it be applied, but it also could serve as the central monitoring tool on the performance of anaerobic digester within various interdependent microbial processes (Alvarado et al., 2014). Potentially common characteristics of methanogenic metabolism in the specialized niche implied that there might be a phylogenetic relationship between methanogens and ANME (Zehnder and Brock, 1979). Based on genomic analysis of AOM in methanogens and ANME, a genetically equivalent enzyme, a counterpart as methyl-coenzyme M (CoM) reductase in methanogenic pathway, might be involved in the reverse methanogenic metabolism (Krüger et al., 2003; Shima and Thauer, 2005). Genome-based research substantiated almost all typical genes involved in methane production in selective groups of ANME, indicating that the potential evolutionary divergence imposed by disparate habitat adaptations may result in related but different methanogenic and methane-consuming metabolic pathways (Hallam et al., 2004).

Combination of observation and metagenetic analysis unraveled a close functional similarity and phylogenetic homology between *mcrA* genes of methanogens and ANME (Hallam et al., 2004). Additionally, the typical polymerase chain reaction (PCR) primers used for detection based on *mcrA* gene of methanogens could also be engaged for ANME detection (Hales et al., 1996; Hallam et al., 2003). It is possible to quantitatively detect the abundance and diversity of methanogens and ANME using *mcrA* gene as a biomarker (Nunoura et al., 2006, 2008).

Aerobic methanotrophic activity in the oxic layer of wetland serves as an important methane sink. It is believed that more than half of methane produced in the anaerobic layers of wetland was consumed in the aerobic layers, for example, rhizosphere and oxidized soil–water interface (Le Mer and Roger, 2001). Genes encoding particulate forms of methane monooxygenase (pMMO) are present in almost all aerobic methanotrophic bacteria (MOB) with minor exception (Theisen and Murrell, 2005) and often used as a biomarker to detect and quantify MOB in the past decades (McDonald and Murrell, 1997). Methane cycling associated microorganisms including methanogens and aerobic methanotrophs in several different environments had been characterized and metabolic relatedness of these two groups was highly connected along eﬄux pathway from deep anoxic layer to upper oxic layer, which could help explain methane flux dynamics in natural environments. For instance, community structures and distribution pattern of *mcrA* and *pmoA* genes-harboring populations vertically from oxygenated upper layer to anoxic deep water layer were assessed in freshwater meromictic Lake Pavin, where *pmoA* gene phylogeny showed that majority of active MOB in the oxic layer of water column were *Methylobacter* with high possibility for consuming nearly all methane from the deeper anoxic layer. In addition, in anoxic sediment of Lake Pavin, hydrogenotrophic and acetotrophic methanogens shared the equivalent existence and could be the reason of active methanogenesis rates, meanwhile, the water column also represented methane production and its methanogenic community was exclusively composed of hydrogentrophs (Biderre-Petit et al., 2011). Another study on spatial-temporal variations of the abundance of *mcrA* and *pmoA* genes together with the 16S rRNA gene of bacteria and archaea was carried out and showed a similar fluctuation from a Japanese wetland and *mcrA* gene abundance negatively correlated with dissolved organic carbon and positively correlated with peat temperature. Moreover, its first attempt to investigate biomass variation in 200 cm depth testified stable and perennial existence of *mcrA* gene in deep layer and suggested that potential effects of peat temperature and peat supply should be taken into concise consideration (Akiyama et al., 2011). In terms of *pmoA* gene abundance in peat bog, nearly anoxic layer under 50 cm depth showed no existence; however, under detectable *pmoA* gene abundance only during spring thaw period in oxic layer suggested *in situ* peat temperature would be the most influential factor and detailed dynamics within these months could be of valuable explanation to methanotrophic process in peat bog environments (Akiyama et al., 2011).

Naturally occurring community of methanogens and ANME assembly and function in wetland area, coastal intertidal zone, pristine ocean, and methane seeps has been reported before. Comprehensive studies on the *mcrA* gene-harboring communities under different environmental conditions and their distributions with other methane cycling microorganisms may provide additional details on niche specificity of methanogens and methanotrophs. A study was initiated attempting to explore biodiversity and community composition pattern of methanogens and ANME in shallow and deep marine sediments from the northern South China Sea (nSCS) and the coastal Mai Po Nature Reserve for a comparative analysis. In the study, the transition of methanogen community structures in shallow marine surface sediments along the gradient from Pearl River Estuary to deep pristine nSCS was ascertained. Moreover, responses of community structures to niche specificity were delineated by analysis of *mcrA* gene-based communities from all the samples in this study.

## MATERIALS AND METHODS

### SAMPLING AND PHYSIOCHEMICAL PROPERTIES

**FIGURE 1:**
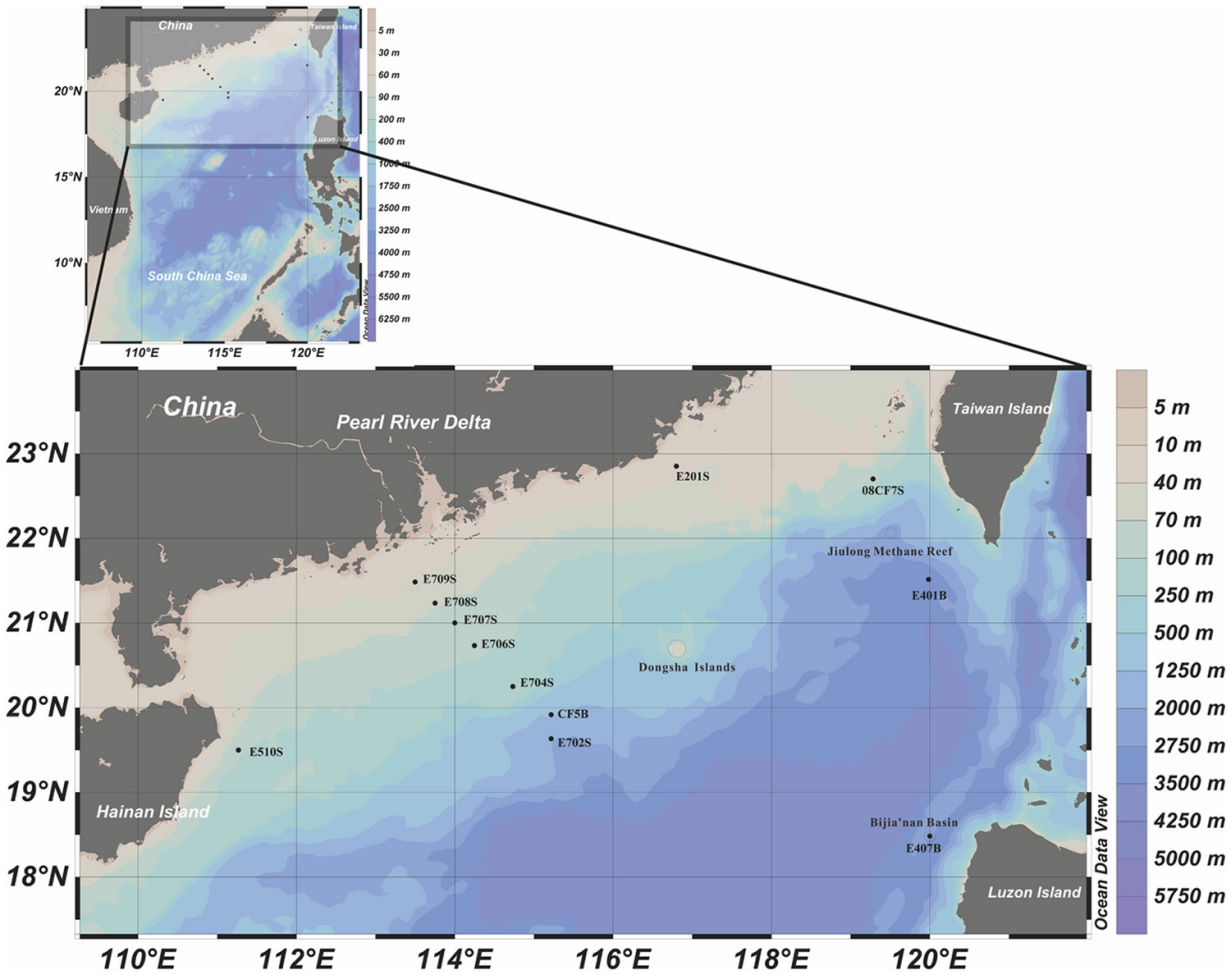
**Geographic locations of sampling sites in the northern South China Sea (nSCS).** This illustration was made with Ocean Data View 4.0 software (). Methane reef location and prospective methane hydrate harboring places were circled with dash lines.

The geographic information of sampling area of this study is summarized in **Figures 1** and S1. Sediments from nSCS were collected during SCS Open Cruise in July 2008 and sediments from intertidal mudflat, mangrove, and reedbed rhizosphere were collected from Mai Po Nature Reserve located in the northwestern New Territory, Hong Kong. Physiochemical parameters of sampling sites from nSCS are shown in **Table 1** according to previous research (Cao et al., 2011a,b). Those samples from Mai Po Nature Reserve were retrieved from our unpublished data and presented in Table S1. Physiochemical parameters of one portion of the samples were immediately measured and the remaining portion was frozen at –20^∘^C for long-term storage. As for samples from Mai Po, pH value, redox value, and water content were obtained after transported back to laboratory within an hour. The remainings were measured according to description elsewhere (Cao et al., 2011c). All samples were assigned labels according to its collected sites individually.

**Table 1:** Physiochemical parameters and location information together with *mcrA* gene diversity and richness indices of sediments collected from South China Sea.

Sampling site	Sampling position	Seawater depth (m)	Temperature (^∘^C)	Depth (mbsf)	pH value (‰)	Salinity	Number of valid sequences	OTU	Coverage	Chao1	Shannon–Wiener index
E401B	21^∘^31N/119^∘^59E	3300	2.5	3.7	NK	34.39	47	15	0.9362	15.9	2.4664
E407B	18^∘^29N/120^∘^00E	1900	2.0∼4.0	1	NK	34.56	48	13	0.8750	23	2.1579
CF5B	19^∘^55N/115^∘^13E	1153	2.0∼4.0	7.5	NK	34.56	47	13	0.9362	12	2.0751
08CF7S	22^∘^70N/119^∘^17E	1301	2.0∼4.0	0∼0.1	NK	NK	46	11	0.9783	10.25	2.0846
E702S	19^∘^38N/115^∘^13E	2370	2.9	0∼0.1	7.5	34.58	42	13	0.9048	15.67	2.3181
E704S	20^∘^15N/114^∘^44E	175	13.5	0∼0.1	7.77	34.57	45	12	0.9111	14.25	2.2733
E706S	20^∘^44N/114^∘^15E	79	18.9	0∼0.1	8.01	34.39	46	7	0.9783	7.5	1.6134
E707S	21^∘^00N/113^∘^60E	80	18.5	0∼0.1	8.08	34.36	47	1	1.0000	–1	0
E708S	21^∘^14N/113^∘^45E	70	19.5	0∼0.1	8.18	34.32	50	5	0.9600	7	0.5837
E709S	21^∘^29N/113^∘^30E	40	21.3	0∼0.1	8.21	34.21	53	10	0.9623	11	1.948
E510S	19^∘^30N/111^∘^16E	100	20.2	0∼0.1	7.54	34.44	50	17	0.8000	67	2.3425
E201S	22^∘^51N/116^∘^48E	30	21.4	0∼0.1	8.2	34.32	46	18	0.8043	49	2.5833

### DNA EXTRACTION AND PCR AMPLIFICATION, CLONING LIBRARY CONSTRUCTION

All genomic DNA of collected samples used in this study were extracted with the SoilMaster DNA Extraction Kit (Epicenter Biotechnologies, Madison, WI, USA) strictly following the manufacturer’s instruction. The extracted genomic DNA was used to run agarose gel electrophoresis with Gel Red (Biotium Inc., USA) to evaluate the integral quality of extraction and preliminary estimation of DNA concentration. Primers for *mcrA* gene were chosen as following: shorter primer pairs including forward primer which encompassed a mixture of primer ME3MF: ATGTCNGGTGGHGTMGGSTTYAC and ME3MF-e: ATGAGCGGTGGTGTCGGTTTCAC with the concentration ratio of 250:1 and reverse primer ME2r’: TCATBGCRTAGTTDGGRTAGT; longer primer pairs including forward primer ME1: GCMATGCARATHGGWATGTC and reverse primer ME2r’ (Narihiro and Sekiguchi, 2011).

The PCR reaction mixture with a final volume of 50 μl contained: 2 μl of template DNA (30∼50 ng/μl), 1 μl of bovine serum albumin aiming to potentiate synthesizing accuracy (100 mg/ml, Roche), 10 μl of 5× GoTaq Flexi Buffer (Promega) and 4 μl of Mg^2+^ (25 mM, Promega), 1.5 μl of dNTPs (2 mM, Invitrogen), 5 μl of each forward and reverse primers (20 μM), and 0.25 μl of GoTaq Flexi polymerase (5 U/μl, Promega). Gradient PCR method was used to optimize the suitable annealing temperature and 59.5^∘^C was chosen as the suitable annealing temperature because it is the lowest temperature to avoid non-specific amplicons and capable of obtaining the highest yield. Double identical PCR reactions were conducted for each sample to obtain more amplicons from potential templates and also minimize arbitrary aberrations from one experiment.

Polymerase chain reaction products of each case were purified with QiagenII Gel Extraction Kit (Qiagen, Hilden, Germany) following the guidance of manufacturer’s instruction. Then, clones were constructed into PMD-18T vector (Takara, Japan). The insert fragment sequences were amplified by M13F and M13R primer pairs, and then delivered for sequencing on a 3730xl DNA analyzer (Applied Biosystems, Foster City, CA, USA).

### QUANTITATIVE PCR

Abundance was measured with q-PCR to detect the copy number of targeted gene in each sample. PCR reagent mixture contained 1 μl of DNA template, 0.5 μl of 100 mg/ml BSA, 1 μl of each primer and 10 μl of SYBR Premix (Roche) in each reaction with a total volume of 20 μl for q-PCR using methodology provided by its manufacturer. For *mcrA* and *pmoA* gene, primer pairs ME3MF+ME3MF-e (250:1), ME2r’ and A189F: GGNGACTGGGACTTCTGG, mb661R: CCGGMGCAACGTCYTTACC (Kolb et al., 2003) were employed to amplify targets. Successive 10 fold dilutions of plasmid of PMD-18T inserted with *mcrA* gene from one clone E704S-53 and *pmoA* gene from one clone of 1B-B sample were operated on the machine to generate a standard curve.

### SEQUENCES AND PHYLOGENETIC ANALYSIS

Obtained sequences were firstly checked to filter out meaningless ones and chimeras. All qualified ones were aligned by Clustal W given by MEGA5.05 together with those from previously reported methanogens and ANME groups archaea retrieved from GenBank to build phylogenetic permutation. Subsequently, MEGA5.05 was used to construct phylogenetic trees as the following default settings: neighbor-joint criterion, *p*-distance model algorithm, bootstrap value 1000 times for resampling. A less related arbitrary sequence should be used as a criterion for out-group to generate rootless phylogenetic tree.

Operational taxonomic units (OTUs) divided in the whole assemblages were gained through implementations of online software Fastgroup II (http://fastgroup.sdsu.edu/; Yu et al., 2006). Meanwhile, OTU rarefaction curves and related non-parametric estimate indices, such as Chao1 and the Shannon diversity index, were also calculated by Fastgroup II based on 5% nucleotide sequence cutoff dissimilarity. Online software, Unifrac, was employed to conduct Principal coordinates analysis (PCoA) and Jackknife environment clusters analyses to evaluate relationships between environmental similarity patterns and phylogenetic structure. Uploaded documents were adjusted to meet the requirement given by the instruction. Relative abundance was depicted via bar chart through Microsoft Excel. Log-normalized heatmap and attached dendrogram was used to show abundance and cluster similar samples across phylogenetic profiles by extracted R script from skiff in CloVR under Rstudio environment (Angiuoli et al., 2011).

As for the relationship between the physiochemical parameters and microbial community composition and abundance, Canonical correspondence analysis (CCA) and Redundancy analysis (RDA) were conducted by the software CANOCO 4.5. Pearson moment correlation analysis was also employed to depict the relationships between physiochemical factors and abundance of *mcrA* gene via Microsoft Excel.

### SEQUENCES ACCESSION NUMBERS

Accession numbers were obtained from GenBank database from KF595310 to KF596336 after depositing *mcrA* gene sequences into NCBI.

## RESULTS

### PHYSICOCHEMICAL CHARACTERISTICS OF THE SEDIMENT SAMPLES

Three major groups of samples in this study can be categorized by the depths and sampling sites: deep South China Sea, shallow South China Sea and coastal Mai Po wetland. There are mangrove, mudflat and reedbed in the coastal wetland. The geographic locations of samples from nSCS are showed in **Figure 1**. E401B, E407B, and CF5B were collected from the subsurface close to Taiwan Island, Luzon Island and center position of nSCS, respectively. E702S, E704S, E706S, E707S, E708S, and E709S formed a gradient line with anthropogenic pollution from the Pearl River Estuary to the nSCS. E702S is regarded as a deep-sea sample and the rest are shallow sea surface samples. E201S is coastal surface sample located east to the Pearl River Delta and E510S is coastal sample near Hainan Island. 08CF7S is a surface sample in the surrounding part of Taiwan Strait. Temperature of different sampling sites in nSCS decreased when the depth increased as shown in **Table 1**.

The intertidal mudflat and mangrove samples were collected at Site 1 and Site 3 in Mai Po Nature Reserve (Figure S1). Surface samples and subsurface samples were collected from a depth of 0–2 and 23–25 cm in mangrove rhizosphere and mudflat in March 2012. In addition, reedbed samples were also collected to a depth of 20 cm. Physiochemical properties of them are shown in Table S1. Generally, surface samples had lower pH, higher nitrate concentration than subsurface samples. Redox potential of surface samples was higher than that of subsurface samples in mangrove while it had a reverse trend in mudflat. Nitrate and ammonium concentration patterns were consistent for Site 1 and Site 3 of intertidal zone and mangrove samples. Reedbed rhizosphere had the lowest redox and highest ammonium among all samples.

### AMPLICONS IN SOUTH CHINA SEA

Two rounds of PCR were engaged to retrieve amplicons from sediments in E401B, E407B, and CF5B using shorter PCR primer pairs (Table S2), successful functional gene clone libraries were obtained for E407B and CF5B. Nested PCR was used to amplify *mcrA* gene of the rest samples and effective PCR products and *mcrA* gene clone libraries from all the 12 SCS samples were established (Table S2). The abundance of *mcrA* gene was below the detection limit when conducting q-PCR assays on samples of SCS. The ubiquitous and low abundance of *mcrA* gene in this study of the nSCS could be confirmed, and 1027 valid sequences were retrieved from 21 heterogeneous samples (**Table 1**).

### ABUNDANCE OF *mcrA* AND *pmoA* GENES IN COASTAL WETLAND

**FIGURE 2:**
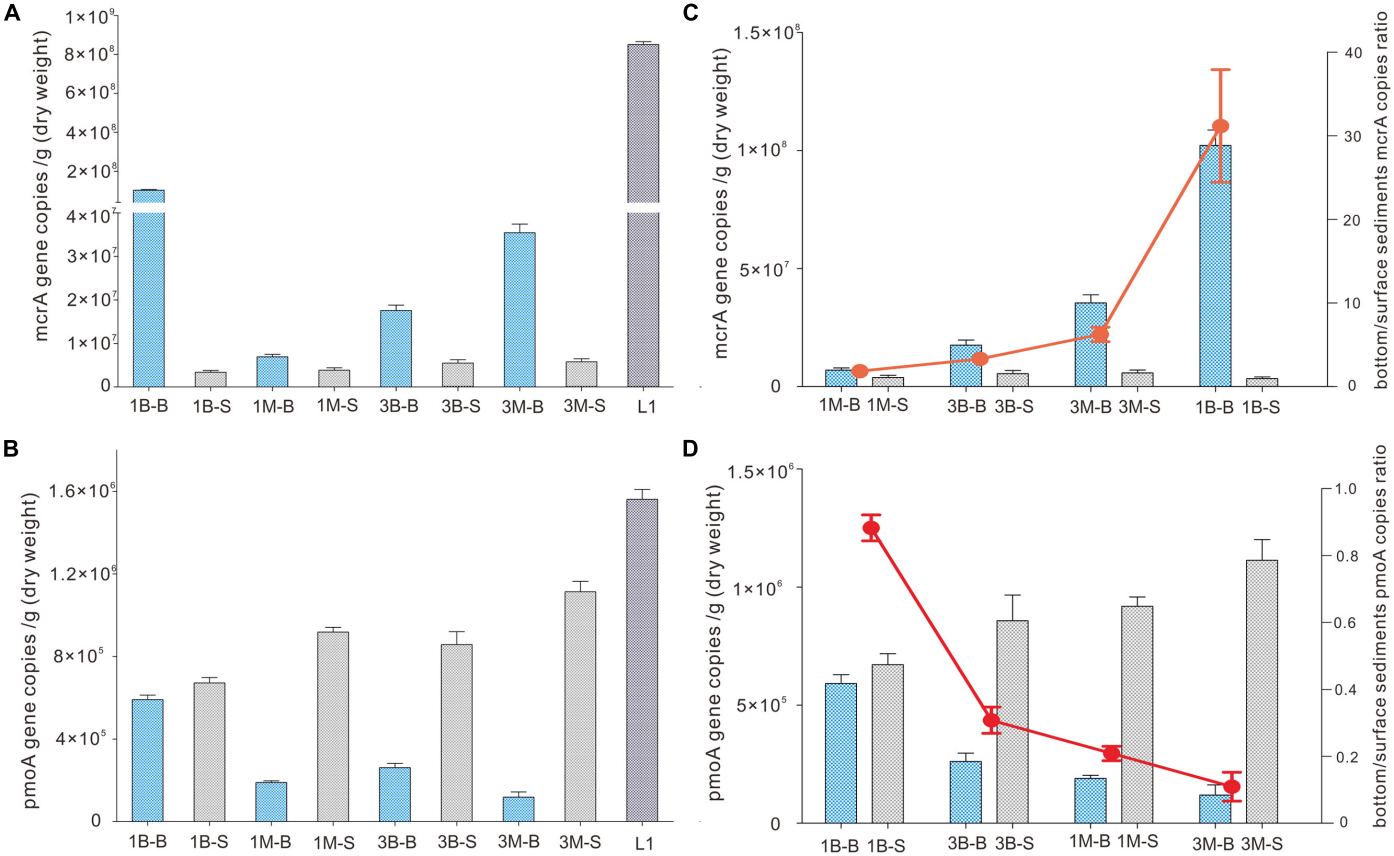
**Abundance of *mcrA* gene **(A)** and *pmoA* gene **(B)** of intertidal mudflat and mangrove sediment samples.** Quantitative PCR gene copies in subsurface (bottom) and surface sediments are expressed in ratio as red dots **(C,D)**. Error bards are vertical lines as standard deviations of three independent experiments.

Abundance of *mcrA* gene was evenly distributed between intertidal mudflat and mangrove samples at Mai Po wetland by qPCR amplification and quantification. It was 3.4∼3.9 × 10^6^ copies per gram dry weight and 5.5∼5.8 × 10^6^ copies per gram dry weight for Site 1 and Site 3 of both layers, respectively (**Figure 2**), but subsurface sediments of sample 1B-B contained higher abundance of *mcrA* gene than the others. Extremely high *mcrA* gene abundance was detected in reedbed sample (L1), consistent with the high organics and less oxygen available with low redox potential. Abundance in subsurface samples had generally 1–3 orders of magnitude higher *mcrA* gene abundance than those in surface samples.

On the other hand, *pmoA* gene distribution and abundance observed from qPCR showed a reverse pattern as that for *mcrA* gene. Abundance of *pmoA* gene was 1–3 orders of magnitude lower than *mcrA* gene in corresponding samples, indicating the dominant role of *mcrA* genes in all samples. Mangrove samples harbored fewer *pmoA* genes than intertidal mudflat samples when examined at the same depth, whereas mangrove surface samples harbored more *pmoA* genes than that of mudflat. The most abundant *pmoA* gene was detected at site L1 among all the samples. The ratio of subsurface gene abundance to surface (B/S ratio) of both *mcrA* gene and *pmoA* gene was mainly determined by gene abundance in subsurface samples because that of surface samples did not show any significant change (**Figures 2C,D**).

### PHYLOGENY OF THE RETRIEVED METHANOGENS AND ANME *mcrA* GENES

Sequences were assigned to all known orders of methanogens except for *Methanopyrales* and *Methanococcales*, indicative of rather diverse community in sediments from nSCS (Figure S2). *Methanomicrobiales* clade was the largest monophyletic one comprising of sequences from Mai Po and South China Sea in two major branches. One was Fen-like cluster, which was named by the predominant sequences previously found in peatland (Galand et al., 2002), containing three clones from mangrove and intertidal zone. This is in accordance with the fact that this cluster is of freshwater origin as both mangrove and intertidal zones from this study could be regarded as low salinity habitat. In the parallel alignment with other samples, clones from mangrove shared their branch with clones from Pearl River Estuary (Jiang et al., 2011) and Marennes-Oleron Bay sediment (Roussel et al., 2009) and those from intertidal mudflat clustered with clones from tidal creek sediments (Edmonds et al., 2008) which were of low salinity environments.

**FIGURE 3:**
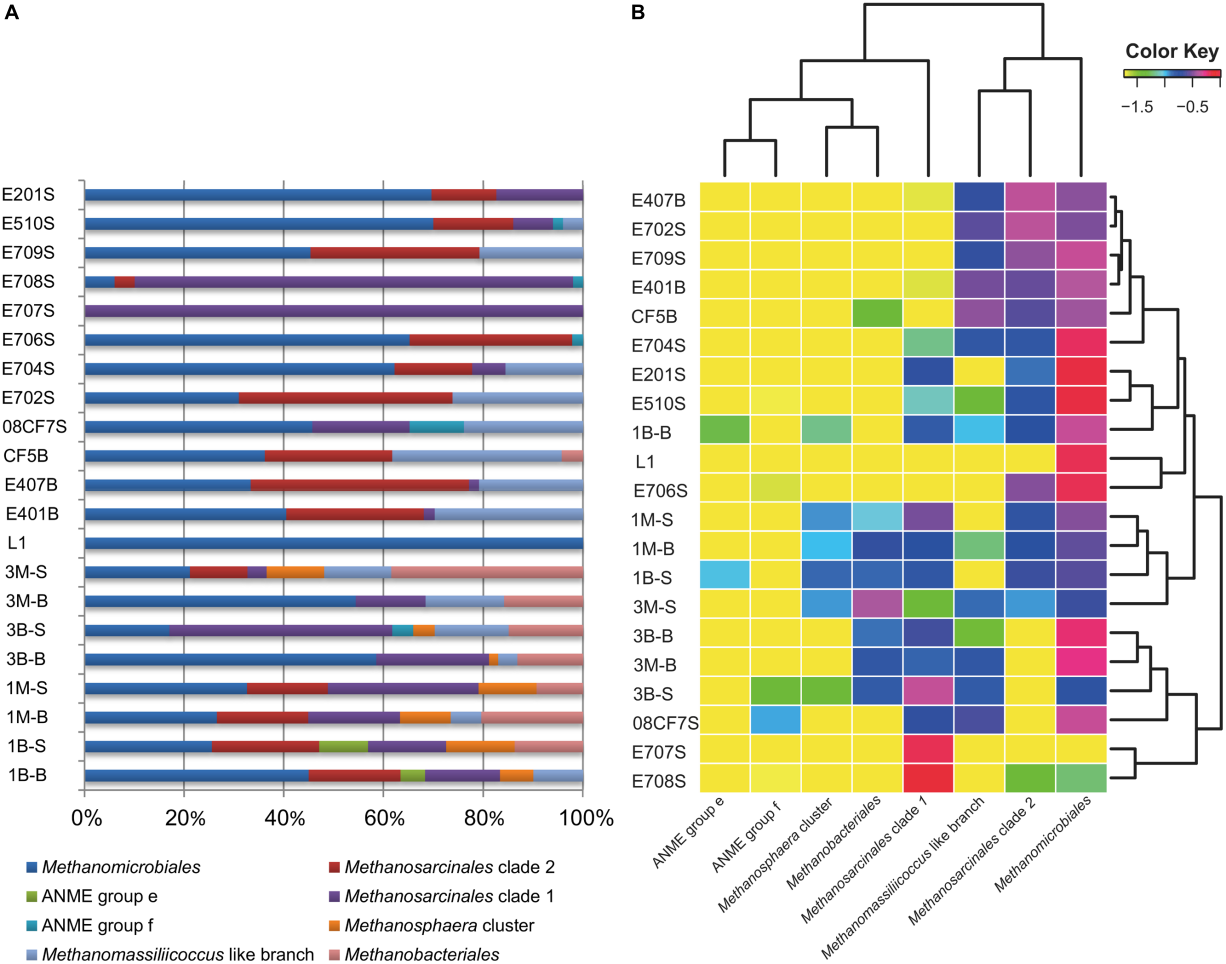
**Visualized *mcrA* gene-harboring archaea composition, abundance and distribution pattern in each sample. (A)** Proportional barchart based on the clone numbers of each group *mcrA* harboring archaea in each sample; **(B)** Log-normalized heatmap made by Rstudio based on the proportion data of clone numbers of each group *mcrA* gene-harboring archaea in each sample. Dendrogram was used to cluster most resembling archaea groups on horizontal axis, most similar sample in respect of community composition on vertical axis. Depicted color key indicated the log-transformed values of abundance percentage values in each sample. This heatmap was generated by extracted R script from skiff module in CloVR software under Rstudio environment.

The other large branch of *Methanomicrobiales* was comprised of sequences from all samples except for E707S. From the percentage diagram of methanogens in each sample (**Figure 3**), *Methanomicrobiales* group occupied a large proportion in samples from Mai Po Nature Reserve and deep-sea sediments. Shallow sea sediments, E708S and E707S showed low diversity of site specificity, dominated by *Methanosarcinales* Clade 2. On the contrary, other shallow sea samples were dominated by *Methanomicrobiales*. The phylogeny of this clade showed rather heterogeneous without noticeable site-specific cluster except for two proposed SCS specific clusters (SCS Clusters 1 and 2) mainly occupied with South China Sea derived sequences.

Sequences in the clade of order *Methanosarcinales* were divided into two parallel clusters. In cluster 1, ANME *mcrA* group f was included. Three kinds of samples all harbored sequences belonging to this anaerobic methanotrophs cluster, which was ANME 3 subtype (Lösekann et al., 2007).

The phylogenetic topology structure had minor changes when incorporating comparable sequences from other niches as suggested in Figure S2, while in both cases the order *Methanosarcinales* could generally be divided into two clusters. Cluster 1 contained a large branch consisting of sequences from Mai Po and shallow SCS sediment samples together with sequences from Marennes-Oleron Bay, pristine tropical mangrove (Taketani et al., 2010), Pearl River Estuary, tidal creek sediment and a distantly located clone from E407B. In cluster 2, most of the sequences were from Mai Po Nature Reserve with five clones from E201S and nine clones from 08CF7S, which were relatively distinct from others, forming two subclusters with sequences from Marennes-Oleron Bay, Shimokita Peninsula subsurface sediment (unpublished data) and brackish lake sediment (Banning et al., 2005) indicating their similarity of ocean origin.

For ANME Group e, both clone library and qPCR data supported that samples from mangrove Site 1 contained Group e ANME (Figures S2 and S3). This mangrove site was the only one contained sequences in the RC-I cluster (Lueders et al., 2001). The nearest sequences were from Pearl River Estuary and tidal creek and all above indicated a unique composition pattern at this mangrove site.

When considering *Methanosarcinales* Clade 2, one distantly located branch and Clusters 3 and 4 constituted this clade. Cluster 3 was composed of SCS sequences and two clones from Mai Po. Few sequences from pristine tropical mangrove and brackish lake sediment were also included in this cluster. Cluster 4 was composed of three different niches of samples. The cultured methanogens were mainly from *Methanosaeta* genus, indicating its distinct classification position in the order *Methanosarcinales*. Clone E709S-49 and five clones from E510S formed a unique cluster deeply branched between *Methanosarcinales* Clade 2. From the blast result, only clones from Jiulong River and basalts samples from Lonar saline soda lake were obtained sharing 90% similarities in amino acid sequences. Regarding to their marine origin, this cluster might be distributed mainly in marine.

**FIGURE 4:**
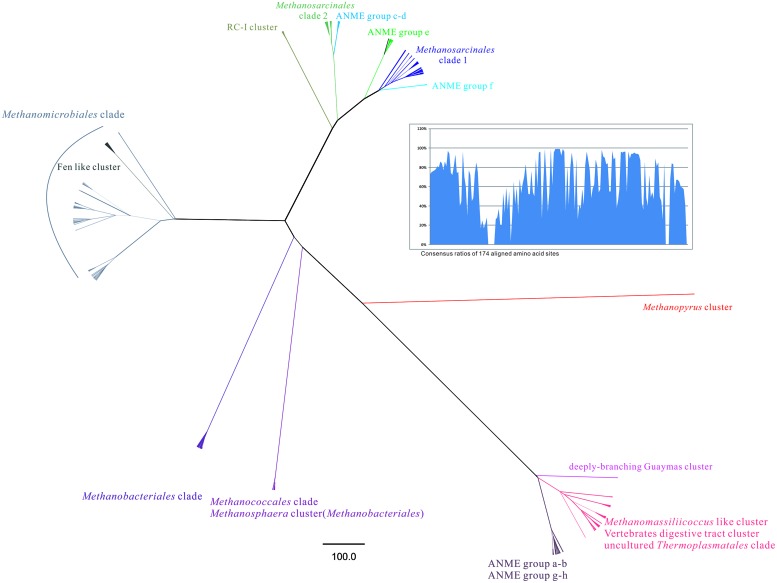
**The radical phylogenetic tree was constructed by the MrBayes 3.2.1 vision based on CIPRES Science Gateway V. 3.3 server ().** In total 800 *mcrA* gene sequences from this study and referred GenBank database was used to build the alignment with 174 amino acid sites including empty sites. The Markov chain Monte Carlo running generation setting was given as 1000000 and 100% majority rule was utilized to conduct a consensus tree. The tree was finally rooted by the out-group of *Methanopyrus* cluster and its branches were transformed by the proportional method. Scale bar was generated to indicate the phylogenetic distance as changes per amino acid position. The line weight of each branch was determined according to posterior probabilities given by Bayesian interference phylogeny. Each known clade was annotated by distinctive color according to the reference sequences. Consensus ratios of 174 aligned amino acid sites were shown by bar chart on the top right part.

The *Methanosphaera* cluster contained one branch exclusively occupied by sequences from Mai Po with high bootstraps value separated from the cultured *Methanosphaera stadtmanae*. In order to reduce the phylogenetic tree of this branch, most similar sequences from GenBank database with similarity values from 92 to 99% were obtained and the results indicated that sequences from this clade have high similarity with those from municipal wastewater sludge digester and sewage. *Methanosphaera* cluster was strictly limited to the substrates of hydrogen and methanol, and mainly found as gastrointestinal tract origin (Liu and Whitman, 2008) and also, together with the discrepancy on phylogenetic position more closely related to *Methanococcales* based on *mcrA* gene (**Figure 4**) other than that based on 16S rRNA gene which falls into the *Methanobacteriales* order and their coccoid shapes other than rod shapes shared by the rest in the order, all makes it mysterious to address its taxonomic relatedness between those two orders above (Luton et al., 2002). Combining with the geographic location of the Mai Po Nature Reserve at the estuarine area of the Pearl River, the evidence of *Methanosphaera* cluster methanogens found in environment might serve as a bioindicator of municipal wastewater input in a similar way as anammox bacteria (Cao et al., 2012; Li et al., 2013).

*Methanomassiliicoccus* genus was newly identified methanogen genus distinct from *Thermoplasmatales*, and sharing the closest relationship with non-methanogenic “*Candidatus* Aciduliprofundum boonei” (Dridi et al., 2012). In this study, some sequences could be categorized into the *Methanomassiliicoccus*-like cluster. Cluster 5 formed a monophyletic clade, consisting of sequences from Mingjiang River (unpublished data) and Jiulong River estuary (Li et al., 2012), two rivers flowing toward East China Sea area. The sequences within Cluster 5 from this study showed 89–97% similarity at amino acid level. Cluster 6 was the most distinctive cluster divided by highly supportive bootstrap value, sharing the highest 86% amino acid similarity with ever known sequences, suggesting unique phylogeny against others.

All the other defined groups were labeled in the whole phylogenetic tree such as newly raised *Thermoplasmatales* clade proposed to be an additional order and other ANME group methanoarchaea. The study performed the most comprehensive illustration on all lineages in the Class *Methanomicrobia*.

Radical phylogenetic tree revealed subclade distance between major groups of methanoarchaea. Three parts of groups were dispersed when rooted by *Methanopyrus* cluster, which were (i) *Methanosarcinales* clade including previously identified ANME Group c to f, grouped with monophyletic RC-I cluster; (ii) *Methanomicrobiales* clade which buried Fen-like cluster inside; (iii) Order *Methanobacteriales*, *Methanococcales,* genus *Methanopyrus*, *Methanomassiliicoccus*, new proposed uncultured *Thermoplasmatales* as seventh order of methanoarchaea and ANME Group a-b, g-h together with unknown deeply branching Guaymas cluster. The consensus ratios distribution pattern of *mcrA* gene coding amino acid reflected certain proportions contributed more on diversification (**Figure 4**).

### DIVERSITY OF METHANOGENS AND ANAEROBIC METHANOTROPHS BY *mcrA* GENE

Sequences obtained in the clone libraries were grouped into 269 OTUs at nucleotide similarity threshold of 95%. Rarefaction curves indicated relatively high coverage indices among all samples of this study (Figure S4). The composition of each group of archaea harboring *mcrA* gene is presented in **Figure 3** together with heatmap of the log-normalized percentage of each group in each sample.

Shannon–Wiener and Chao1 indices were given together with OTU numbers and valid sequence numbers (**Tables 1** and S1). Comparisons between each sample with diversity indices showed that Shannon–Wiener and Chao1 indices were higher in Mai Po samples (mean value ca. 14.8 and 2.3) than in marine samples except for E201S and E510S samples (mean value ca. 11.6 and 1.8; Figure S5). The highest Shannon–Wiener index values were observed with shallow marine samples E201S and E510S among all marine samples. Meanwhile, along the gradient from Pearl River Estuary to nSCS, E706, E707, E708, and E709 samples showed relatively low richness and evenness compared with other marine samples. However, coastal reedbed rhizosphere showed low richness and evenness values among freshwater samples (Figure S5). Lower Shannon–Wiener indices of E707, E708, and L1 samples were possibly due to the predominant species occupied resulting in uneven distribution of each *mcrA* clone library (**Figure 3**). In the Mai Po Nature Reserve, the surface samples harbored more diverse and even *mcrA* communities than subsurface samples from both intertidal sediments and mangrove.

### *mcrA* GENE-HARBORING COMMUNITY STRUCTURE AND CLASSIFICATION

**FIGURE 5:**
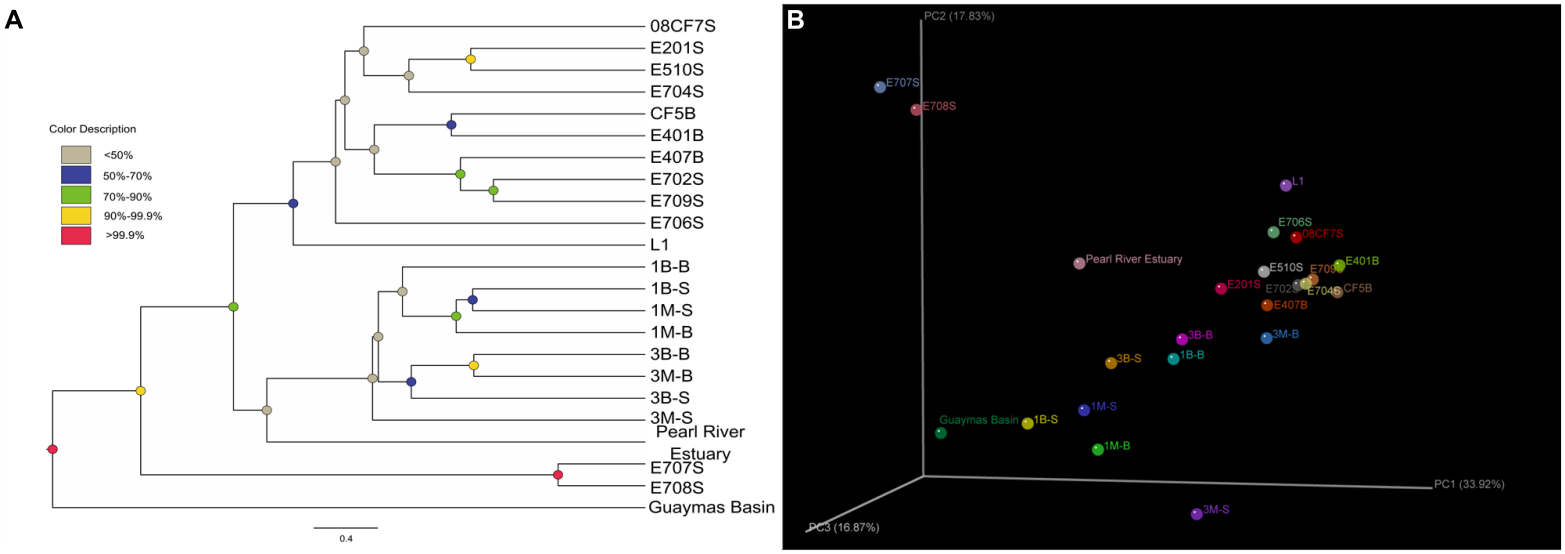
**(A)** Weighted Jackknife sample cluster analysis was conducted by setting the minimum sample counts as 42, permutation numbers as 1000. Finally, the clustering results were shown in **(A)**. Different node colors showed different confidence values of each cluster; **(B)** Weighted 3D PCoA ordination diagram was made by the Fast Unifrac online software package and then illuminated as 3D scatter plots diagram by KiNG 2.21 software () with principal coordinate explanation valued labeled.

The Unifrac-based PCoA was carried out to delineate any differences of environmental heterogeneity by analyzing *mcrA* gene phylogeny among all samples. The weighted Unifrac-based Jackknife samples clustering method suggested the community relationship based on the permutation method to show the confidence at the nodes recovered (**Figure 5**). For E707S and E708S, they located along the PC2 axis separated from others. Their *mcrA* gene communities were dominated by *Methanosarcinales* Clade 2 and showed low diversity value and uneven distribution pattern. The Mai Po samples grouped less concentrated than the marine samples, indicative of relatively more diverse pattern showing in the coastal ecosystem. Pearl River Estuary sample was grouped with Mai Po samples in Jackknife samples cluster, suggesting closer relatedness, while *mcrA* gene community in Pearl River Estuary represented a transition between Mai Po and nSCS area. Reedbed rhizosphere sample was separated from other Mai Po samples and its community structure was classified more closely to marine samples on account of both weighted PCoA and Jackknife sample clustering analysis.

### DIVERSITY AND DISTRIBUTION OF *mcrA* GENE IN WETLAND

**FIGURE 6:**
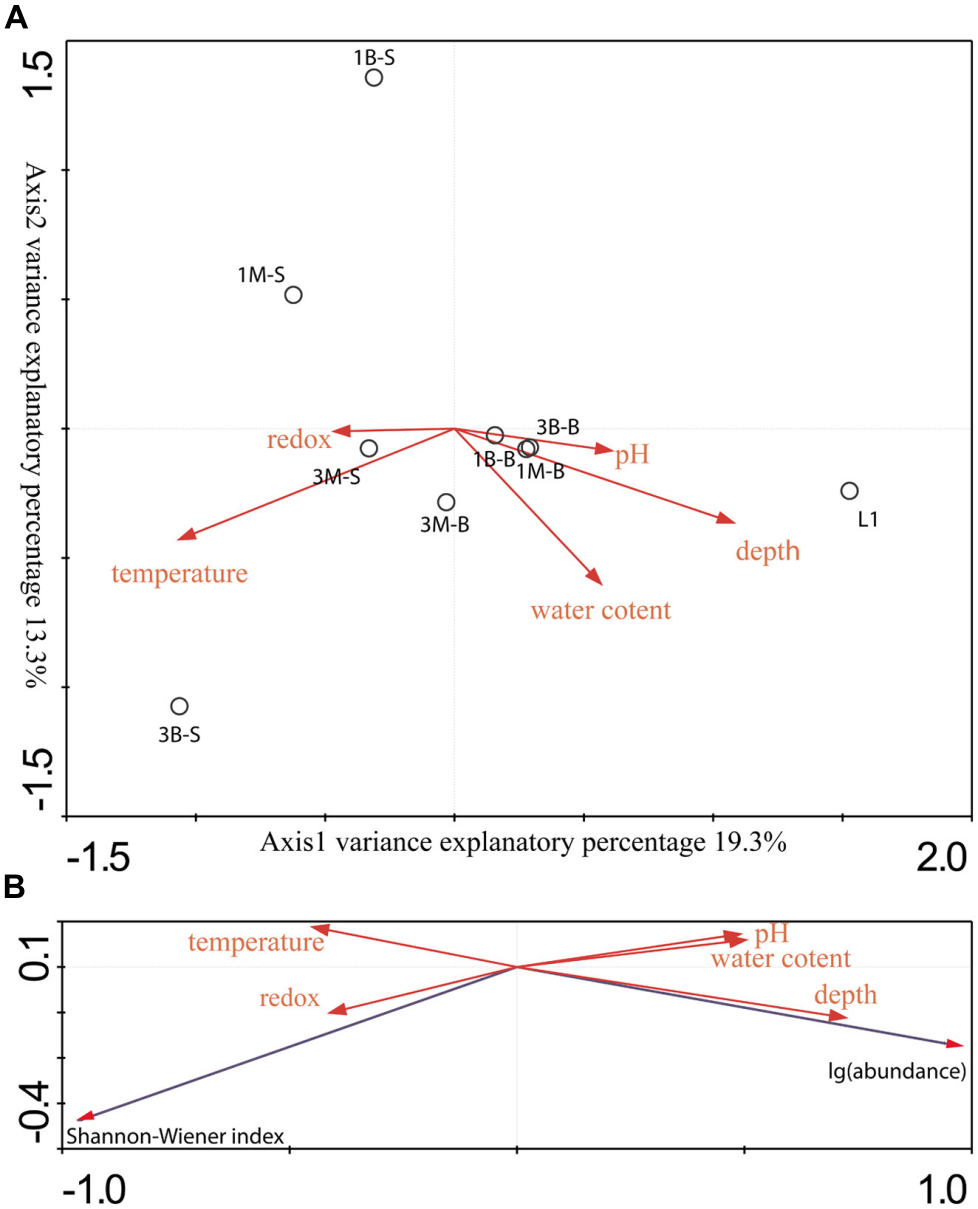
**(A)** Gradient analysis diagram was drawn by direct Canonical Correlation Analysis method and biplot scaling type by 1000 times setting of Monte-Carlo permutation test to analyze the relationship between sample groups and environmental factors. **(B)** Gradient analysis was conducted by redundancy analysis (RDA) method and biplot scaling type by 1000 times setting of Monte-Carlo permutation test to show connection between richness and abundance data and environmental factors.

Two CCA dimensions represented two major axes which yielded the most variance explanatory percentage (32.6%) between *mcrA* genes containing archaeal group composition (**Figure 3**) and environment variables (**Figure 6A**). And axis 1 together with axis 2 accounted for 77.9% cumulative variance percentage of *mcrA* containing archaeal groups and environments. Subsurface samples and surface samples from both mangrove and intertidal mudflat were relatively apart and formed two assemblages according along axis 1. Coordinates of subsurface samples showed in a compact manner, while surface samples scatter along axis 2. L1 sample was separately located indicating different distribution pattern against other sites. Depth and pH values were positively correlated with axis 1, serving as dominating variables which influenced subsurface microbial community. Surface sediment would be more susceptible to perturbations of natural and anthropogenic activities, resulting in alterative physiochemical environmental conditions which alter microbial community. Redox potential values were negatively correlated with depth (showing –0.44 correlative coefficient), and pH values changes were concomitant with the increasing of depth (showing 0.48 correlative coefficient). The three parameters mentioned above showed closed relationship with community structures of these two assemblages, suggesting the stratified environmental condition between the subsurface and surface largely influenced the community composition. L1 was collected from reedbed with abundant organic matter and anaerobic condition possessing both methanogenesis and methane-utilization. Its methanogenic community obtained unique composition and abundance pattern distinguished from mangrove and intertidal zones according to this research. No noticeable parameters in this study were found to account for its distribution in the CCA ordination plots, whereas, considerably large humic compounds, anoxic and low redox potential would be certain factors affecting its richness and uniqueness of methane cycling microorganisms. More comprehensive physiochemical parameters detecting and analyzing model should be elaborated to tell the accurate shaping effects under different environmental conditions.

Regarding to the RDA ordination plots (**Figure 6B**), depth values were the most influential effects on the abundance of *mcrA* gene abundances. All surface samples from different locations obtained low abundance. Additionally, Pearson moment correlation analysis indicated that OTU numbers and abundance were positively correlated with depth significantly (Table S3). Meanwhile, Shannon–Wiener index positively related with redox values.

## DISCUSSION

### COMMUNITY STRUCTURE AND METHANOARCHAEA IN MARINE SAMPLES

Three subsurface samples from nSCS (E401B, E407B, CF5B) belonging to the deep sea were clustered together according to Jackknife cluster analysis (**Figure 5**), consistent with their similarity in major community compositions (**Figure 3**). Subsurface samples were grouped together despite of their different geographic locations. E407B with a relatively shallow depth below sea floor separated slightly from the other two subsurface samples, but close to surface samples, representing an intermediate between two sampling depths. This might contribute to evidence on methanoarchaea distribution affected by depth below seafloor.

Deep-sea surface sample E702S and marginal coastal sea surface sample E709S were grouped closely with subsurface samples with similar community composition. E702S was located in the slope of the continental shelf to pristine deep sea, while E709S was the nearest sampling site to Pearl River Delta, representing a more terrestrially affected environment. Unlike the obvious community transition along the slope from Pearl River Delta to nSCS deep sea area on ammonia oxidizers (ammonia-oxidizing archaea and beta ammonia-oxidizing bacteria; Cao et al., 2011a), E702S and E709S shared less similarity in water temperature, depth and pH values, however, grouped the closest compared with others, suggesting that methanoarchaea communities were not as responsive as ammonia oxidizers to decrease from coast to ocean along the continental shelf.

Samples E201S and E510S were coastal surface samples located in distant geographic points, but they shared the largest similarity with each other. And E704S was also from shallow marginal coastal sea surface, grouped together with the above two samples. Another deep-sea surface sample 08CF7S was characterized as an out-group in surroundings of this clade. This phenomenon could be interpreted that environments divide by surface and subsurface layers share resembling conditions which differentiate community groups rather than the actual geographical locations.

There was an evidence that biogenic originated methane hydrates have been discovered from Shenhu area which located at the southeast of Dongsha Islands (Wu et al., 2011). According to the previous definition (Zhong et al., 2006), site E702S and CF5B were located in this area, which harbored worm tube containing carbonate nodules as evidence of moderate micro-gas venting. Meanwhile, Jiulong methane reef near Taiwan Island and Bijia’nan Basin near Luzon Island were found to be potential locations harboring gas hydrate (Youhai et al., 2001; Chen et al., 2005; Han et al., 2008). Based on above information, maps illuminating prospective gas hydrate reservoir area were elaborated (Zhou et al., 2009; Dang et al., 2013). Hence, sites E401B and E407B could be possibly ones for prospective gas hydrates due to their locations in these areas. Other information of methane hydrate and methane seep distribution feature in the nSCS area indicated that low supply of methane was common and occurred recently at those prospective methane hydrate areas (Zhong et al., 2006; Wu et al., 2011). Taken all these into consideration, all prospective methane hydrate samples could be grouped as potential methane rich environments and actually clustered close to form a clade in Jackknife cluster tree expect E709S site, leading to a proposed hypothesis that relatively higher methane concentration might be the result of potential biogenic gas or hydrate form’s methane caused by featured methanogenic archaeal communities, which mainly depend on categories and abundance of accessible methanogenic substrates. Accordingly, E709S sampling site was not yet physically testified but highly presumed to be methane rich area because it has close affinity with E702S in community structure.

Site E706S was distinguished from other marine samples and this may be the result of its niche specific environmental conditions. Community composition in freshwater sample L1 showed its uniqueness and uneven feature of low diversity value and predominated by archaea order *Methanomicrobiales* when compared with Mai Po samples. As far as the other two marine samples E707S and E708S were concerned, they were composed majorly by *Methanosarcinales* clade 1 group archaea, and demonstrated the lowest diversity indices among all marine samples. Those unique features made the two samples forming a distinctive cluster separated from the rest marine samples in the Jackknife cluster diagram. From the other shallow samples’ community composition, we could find that *Methanosarcinales* clade 1 and clade 2 together constituted the majority. The community transition of these two marine samples could be a result of substrate changes to the dominated methyl containing compounds which were thought to be mainly consumed by species under *Methanosarcinales* clade 1 (The most related known genus in GenBank database is *Methanococcoides*, which entirely depend on methylotrophic nutrition; Liu and Whitman, 2008).

In attempt to detect amplify *mcrA* genes or methanogen related 16S rRNA genes from deep sea drilling samples, *Methanobacteriales* and *Methanosarcinales* species have been discovered, however, as stated methanogens could be a very small population located in the hydrate-bearing sites at Cascadia and Peru Margins (Inagaki et al., 2006). Moreover, the small sized of methanogen groups in archaeal clones also resulted in the undetectable of methanogens in Nankai Trough (Reed et al., 2002), while evidence of a new isolated methanogen species from Nankai Trough in the same time suggested the clear existence (Mikucki et al., 2003). Amplification method should be responsive for those deviations especially for small groups of methanogens (Reed et al., 2002; Hu et al., 2011).

Overall, in nSCS, widespread methanogens were retrieved in shallow and deep sea sediments. The majority of methanogen in those samples by *mcrA* gene contained: *Methanomicrobiales, Methanosarcinales* clade 1,2 and *Methanomassiliicoccus*-like groups.

*Methanomassiliicoccus* was firstly found in human gut microbiome (Dridi et al., 2012; Borrel et al., 2013), while, in this study, clusters of *Methanomassiliicoccus*-like groups with high similarity composing of nSCS sediments and estuaries toward East China Sea gave us new insights of these important methanogens. To our knowledge, this is the first time to comprehensively analyze methanoarchaea community composition from marine sediment samples.

### COMMUNITY STRUCTURE AND DIVERSITY OF METHANOARCHAEA IN WETLAND

Mai Po Nature Reserve samples were collected by the strategy of sampling sites, sediment types, and different depth layers. From the physiochemical parameters detected based on this strategy, pH values of mangrove samples were always lower than those of mudflat; those of surface samples were always lower than those of subsurface. The main reason might be the effects of seawater. It is usually thought that sea water is slightly alkaline, and intertidal mudflat might be more influenced by seawater than mangrove field, because intertidal mudflat is without any plantations and near coast. In addition, overlying layers could be more dynamically influenced by rainwater, so subsurface samples were more alkaline than the surface ones.

In general view, mangrove samples obtained higher redox potentials than mudflat samples. This could be resulting from respiration effect of mangrove roots which will transport plenty of oxygen into rhizosphere. At the same time, NH_4_^+^ concentration was also testified to be much less in mangrove subsurface samples than others. It might be due to absorption activity of mangrove roots and associated nitrifying microbes (Glaser et al., 2010; Wang et al., 2013). In addition, reedbed rhizosphere sample was also included in this study, which was alkaline, anaerobic and had a high concentration of NH_4_^+^. Previous studies claimed that reedbed rhizospheric methane oxidation should account for 80% of total oxidation in the emergent plant period but with relative lower efficiency than surface oxidation, indicating that reedbed rhizosphere could serve as a unique environment of methane oxidation process in low oxygen condition (van der Nat and Middelburg, 1998). Different physiochemical environments distinguished by three parallel sampling factors covered major compositions in Mai Po Nature Reserve, which will surely reflect different *mcrA* gene community and abundance among this area and help to ascertain which factors counted for the differences.

From the analysis of Jackknife clustering, we could easily find that samples were majorly separated into two parts in accordance with the sample sites: site 1 and site 3. As for each site, it is also obvious to point out that different layer harbored more similar communities. For example, site 1 cluster was composed with 1B-S and 1M-S groups and surrounded by subsurface samples. Site 3 cluster was comprised of the group of 3B-B and 3M-B firstly, and then with additional surrounded by surface samples. This phenomenon suggested that different sampling site conditions influenced the community most and different depths were the second important factor.

Surface samples were the top layer and influenced by variable environments, such as those imposed by intertidal ecology and occasional weather change and bioturbation. CCA showed that surface samples represented a more dispersed pattern resulting from relatively more variable environmental conditions in the surface (**Figure 6A**). This could interpret higher methanoarchaea community diversity values observed in the surface samples both in mangrove and intertidal mudflat samples. And on the other hand, subsurface layer samples usually acquired low oxygen and low redox potential, especially in the reedbed rhizosphere sample, representing much less diversity patterns.

In contrast to our results, previous investigation on *mcrA* gene abundance showed that the abundance ranging from 2.75 × 10^5^ copies per gram sediments to 1.83 × 10^6^ copies per gram sediments in nSCS (Dang et al., 2013). But another attempt in detecting the *mcrA* gene abundance in subseafloor sediments from northern and southern SCS obtained negative results even though different PCR annealing temperatures were tested (Hu et al., 2011).

With respect to *mcrA* abundance, *mcrA* gene copies were much higher in subsurface samples than surface samples. There were other references indicated that MLf and MLr primer pairs from Luton et al. (2002) could also amplify the isoenzyme encoding gene *mrtA* of *Metahanobacteriales* and *Methanococcales* (Friedrich, 2005; Nettmann et al., 2008), however, due to the fact that no *mrtA* genes appeared in the phylogenetic reconstruction of *mcrA* gene amplified by the same ME3MF and ME3MF-e and ME2r’ primer pairs, this possibility of non-specific amplification could be rather small. At the same time, litter relatedness between *mcrA* gene abundance and sample type was found suggesting that no noticeable feature on the abundance pattern shared by the two sampling sites. This is in line with community character indicated by Jackknife clustering analysis. Pearson moment correlation analysis suggested that depth was positively correlated with log_10_ value of abundance and negatively correlated with OTU numbers. Taken all together, those above could be a consequence of distinctive niche physiochemical conditions at each site. Lower layer had low oxygen concentration and low redox potential in intertidal mudflat sediments, which was suitable for anoxic microorganisms such as methanoarchaea to grow. At the same time, lower layer environment may also activate growth of some predominant groups of methanoarchaea which in turn attenuate its diversity profile (this phenomenon was testified previously in **Figure 3**. *Methanomicrobiales* generally occupied around 50% of the entire community in intertidal mudflat subsurface samples). As for the lower layer mangrove samples, they were sampled from rhizosphere of mangroves. Exudates and detritus from mangrove roots could supply substrates for methane generation and relatively high oxygen condition reversely limited the proliferation of methanoarchaea. As a result, it could be more dynamic in terms of the *mcrA* abundance in mangrove rhizosphere.

### METHANOTROPHS ABUNDANCE IN DIFFERENT DEPTHS

As for *pmoA* abundance information, depth and sediment site both affected the amount. Mangrove samples harbored more *pmoA* gene copies than intertidal mudflat, and surface samples harbored more *pmoA* gene copies than subsurface samples. These patterns were shared, irrespective of sampling sites. It should be noted that subsurface samples harbored fewer *pmoA* gene copies, but the surface samples had more abundant *pmoA* gene at the same site. This could be the first report in consideration of abundance distribution of the *pmoA* gene in coastal wetland in relation to depth as far as the up-to-date references concerned. Methane eﬄux and uptake should be in accordance to flux transport from subsurface to surface, and the total net methane flux differences could be relatively insignificant in mangrove and intertidal mudflat on the basis of former study (Chen et al., 2010) and methane production will be comparatively stable from the same sampling sites which share similar spatial and temporal variances (Allen et al., 2007). As a result, whole subsurface and surface methanotrophic community abundance will be also at a stable level if there is no considerable discrepancy in methane oxidation activity between cells, meaning that higher *pmoA* gene abundance in surface than subsurface should be evident.

Further studies are necessary to detect *in situ* methane oxidation rate because this phenomenon could be common in coastal wetland irrespective of sediment type. Abundance of *pmoA* gene was also detected in peat soil samples collect from different rhizosphere from Zogie wetland of Tibetan plate (Yun et al., 2012), ranging from 10^7^∼10^8^ gene copies and transcripts copies per gram dry soil. Our results from Mai Po Nature Reserve were comparable with other quantitative data of *pmoA* gene in non-vegetation peat soil from Zogie wetland (Yun et al., 2010), flooded rice field (Kolb et al., 2003) and forest soil (Kolb et al., 2005), ranging in the level of 10^6^ copies per gram dry soil.

### DISTRIBUTION OF ANME IN nSCS AND WETLAND

In the ANME f group divided by the *mcrA* phylogeny which is buried in the large cluster of *Methanosarcinales* clade 1, shallow and deep marine samples together with Mai Po samples all contributed minor parts of sequences in this clade. Whereas, the ANME e group was only detected in the Mai Po samples, specifically in the 1B site surface and subsurface layers (Figure S3). Previously study confirmed the evidence that ANME-1 and ANME-2a were divided according to 16S rRNA gene found in the Pearl River Estuary (Jiang et al., 2011), while ANME-2a predominately in the ANME group. Jiulong River estuary sediments were also investigated in the 16S rRNA, 16S rRNA gene, and *mcrA* gene based phylogeny, testifying ANME-2a was also the representative ANME group despite of its minor composition in the whole archaeal communities (Li et al., 2012).

Our results indicated that in the Mai Po mangrove marsh, ANME e (ANME-2a) group was the major ANME group based on retrieved clone numbers and semi-quantitative PCR (supplemental materials) while small proportion of ANME f (ANME-3) was also detected, implying multiple phylotypes of ANME participating in AOM process. On the other hand, ANME f (ANME-3) sequences were detected from shallow and deep marine samples from nSCS with few clones, directly verifying the qPCR results of ANME f group distribution in nSCS (Dang et al., 2013). First *mcrA* gene evidences of ANME f which are congruent phylogenetically with 16S rRNA genes in ANME-3 was unveiled in HMMV sediments under the sulfide-oxidizer mats (Niemann et al., 2006). Other single findings of ANME-3 were also found in cold seeps with high gas hydrate or active seepage of gas under the predominated population of ANME-1 and ANME-2 (Orphan et al., 2001; Knittel et al., 2005) and sporadic 16S rRNA gene evidences were also shown in the previous studied samples such as incubated cold methane seep samples from Monterey Canyon, Eel River Basin and sulfide chimney in Mothra Vent Field on the Juan de Fuca Ridge (Knittel et al., 2005).

Here, we gave an indication of ANME-3 group archaea existence in marine sedimentary samples and coastal intertidal mudflat, which normally believed to harbor moderate or low AOM rate (Knittel and Boetius, 2009). SMTZ is a main specific niche for marine sediments and coastal water column, where methane produced below and synergistic produced sulfate or ambient sulfate overlapped in this zone, resulting in minimum yield of energy in AOM. In contrast to those in methane seeps with high AOM rate, these groups of ANME-3 form adaptive mechanism under energetically less favorable conditions (Knittel and Boetius, 2009). More available PCR primer sets could be applied to detect minor ANME *mcrA* genes within methanogen community, so that more detail analysis could be delineated (Zhou et al., 2014).

Based on methyl-coenzyme M reductase alpha subunit (*mcrA*) genes, community and diversity patterns in nSCS marine sediments and Mai Po Nature Reserve have been revealed and a comparison to other environments was made. In general, Mai Po wetland sediments have higher diversity than marine sediments, but the community diversity was not significantly correlated with the depth of the marine samples. In Mai Po wetland, the surface layer showed higher diversity but lower abundance than the subsurface layer. Additionally, conditions at different layers exercise effects on the *mcrA* gene diversities and community composition. We also measured the quantity of aerobic methanotrophs by means of detection of *pmoA* gene abundance, and found that mangrove samples harbors more *pmoA* gene copies than intertidal mudflat samples in the surface layer while fewer *pmoA* gene copies in the subsurface layer. Besides, the total *pmoA* gene abundance was relatively stable when adding surface and subsurface samples together. However, due to the possible presence of *mcrA* and *pmoA* genes could be inactive in methane cycling microorganisms, analysis on transcript abundance of each gene in the future should be carried out to reflect their real activity (Alvarado et al., 2014).

To summarize, the marine and coastal wetland served as energetic and variable environmental habitats for activity of methanoarchaea with different distribution patterns and functional groups. Their relationship between *in situ* physiochemical parameters and community composition and geographic distribution still needs higher resolution measurement technique and strategies to unravel. However, our results are indicative of substrate compounds and depth distinction may effectively lead to structural and functional differences.

## Conflict of Interest Statement

The authors declare that the research was conducted in the absence of any commercial or financial relationships that could be construed as a potential conflict of interest.
