# Supplementary material for: Analysis of methane-producing and metabolizing archaeal and bacterial communities in sediments of the northern South China Sea and coastal Mai Po Nature Reserve revealed by PCR amplification of mcrA and pmoA genes
Source: Front Microbiol. 2015 Feb 17;5:789. doi: 10.3389/fmicb.2014.00789 (PMC4343527; doi:10.3389/fmicb.2014.00789)
Supplement: Supplementary file 1 [file Presentation_1.PDF]

Supplementary materials

Comparison of Communities of both Methane-producing and Metabolizing Archaea and Bacteria in Sediments between the Northern South China Sea and Coastal Mai Po Nature Reserve Revealed by PCR Amplification of *mcrA* and *pmoA* Genes

Zhou Zhichao[1], Jing Chen[1], Huiluo Cao[2], Ping Han[1], Ji-Dong Gu[1]*

[1] Laboratory of Environmental Microbiology and Toxicology, School of Biological Sciences, The University of Hong Kong, Pokfulam Road, Hong Kong SAR, Hong Kong, PR China

[2] Division of Life Science, Hong Kong University of Science and Technology, Clear Water Bay, Hong Kong SAR, Hong Kong, PR China

*Corresponding author

Dr. Ji-Dong Gu
School of Biological Sciences
Kadoorie Biological Sciences Building
Pokfulam Road, Hong Kong SAR, PR China
email:jdgu@hku.hk

Table S1. Physiochemical parameters and location information together with *mcrA* gene diversity and richness indices of sediment samples collected from Mai Po Natural Reserve

Site	Location	pH	Redox (mv)	Temperature (°C)	Water content (%)	Ammonium (μm/kg dry soil)	Nitrite (μm/kg dry soil)	Nitrate (μm/kg dry soil)	Number of valid sequences	OTU	Coverage	Chao1	Shannon-Wiener index
1B-B (Mudflat Subsurface)	N22 °29' 40.7",	7.41	-178.6	22.7	31.74	40.01	6.11	3.40	60	10	0.9833	10.5	2.072
1B-S (Mudflat Surface)	E114 °01' 42.1"	7.27	-202.0	23.7	29.51	17.13	5.62	10.52	51	17	0.8824	28.5	2.5994
1M-B (Mangrove Subsurface)	N22 °29' 39.8",	6.86	-161.4	21.9	37.54	0.51	9.00	10.26	49	17	0.9184	16.5	2.5437
1M-S (Mangrove Surface)	E114 °01' 43.6"	6.16	-49.3	22.5	38.30	9.73	8.06	42.10	43	21	0.8605	20.25	2.796
3B-B (Mudflat Subsurface)	N22 °29' 56.8",	7.66	-152.6	22.5	34.85	48.72	9.48	22.48	53	16	0.9245	14.9	2.1917
3B-S (Mudflat Surface)	E114 °01' 39.0"	7.13	-181.2	27.6	50.86	17.06	15.57	163.07	47	18	0.8511	20.6	2.5961
3M-B (Mangrove Subsurface)	N22 °29' 55.7",	6.80	-137.0	22.3	32.94	0.07	6.94	4.94	57	11	0.9298	9.25	1.9616
3M-S (Mangrove Surface)	E114 °01' 41.2"	6.49	38.5	22.1	54.17	13.11	13.96	60.03	52	18	0.9038	14.125	2.5038
L1 (Reedbed Sediment)	N22 °29' 34.5", E114 °02' 42.1"	7.31	-192.0	21.1	70.32	365.80	31.56	76.76	48	6	0.9583	-1	1.0175

Data retrieved from unpublished paper from Jing Chen, Ji-Dong Gu et al.

Table S2. Thermal cycling and PCR rounds settings used in each sample amplifying *mcrA* gene amplicons

**Sample Name**	**E407B**	**CF5B**	**E401B**	**E702S**	**E704S**	**E706S**	**E708S**	**E709S**	**E510S**	**08CF7S**	**E201S**	**E707S**
PCR method	2 rounds<br>ME3MF+ME3MF-e(250:1);ME2r' [40cycles]<br>95℃ 2min<br>40cycles(95℃ 30s, 59.5℃ 30s, 72℃ 40s)<br>hold 72℃ for 10min		1 round ME1;ME2r' [35cycles]<br>95℃ 2min<br>35cycles(95℃ 30s, 59.5℃ 30s, 72℃ 50s)<br>hold 72℃ for 10min<br>1 round ME3MF+ME3MF-e(250:1);ME2r' [40cycles]<br>1 round short-PCR ME3MF+ME3MF-e(250:1);ME2r'[15cycles]									1 round ME1,ME2r' [35cycles]<br>1 round ME3MF+ME3MF-e(250:1);ME2r'[40cycles]

**Sample Name**	**1B-B**	**1B-S**	**1M-B**	**1M-S**	**3B-B**	**3B-S**	**3M-B**	**3M-S**	**L1**
PCR method	1 round ME3MF+ME3MF-e(250:1);ME2r' [35cycles]								

Table S3. Pearson moment correlation analysis of potential co-variability between physiochemical parameters potential and diversity and abundance of *mcrA* gene harboring microorganisms

	OTU	Log(abundance)[1]	Shannon-Wiener index
pH	-0.51	0.45	-0.49
Redox	0.47	-0.37	0.43
Temperature	0.39	-0.44	0.40
Water content	-0.32	0.46	-0.50
Depth[2]	-0.71*	0.70*	-0.66

This Pearson moment correlation analysis was based on the substituting estimation on variance relationship between physiochemical parameters and microbial composition and abundance information of site-specific environmental samples. The correlation values (represented by the r) were calculated by the equation:

$$r = \frac{\sum_{i=1}^{n}(X_i - \overline{X})(Y_i - \overline{Y})}{\sqrt{\sum_{i=1}^{n}(X_i - \overline{X})^2}\sqrt{\sum_{i=1}^{n}(Y_i - \overline{Y})^2}}$$

. Asterisked values meant the pearson correlation coefficients are significant (P<0.05), determined by Microsoft Excel double tails TDIST calculation. The t values were deduced from the formula: $t = \frac{r}{\sqrt{\frac{1 - r^2}{n - 2}}}$ (n referring to the sample numbers).

1. "lg (abundance)" was $\log_{10}$ value of each abundance amount.
2. Depth information was normalized as defined this: subsurface samples were 20 centimeters, surface samples were 2 centimeters.

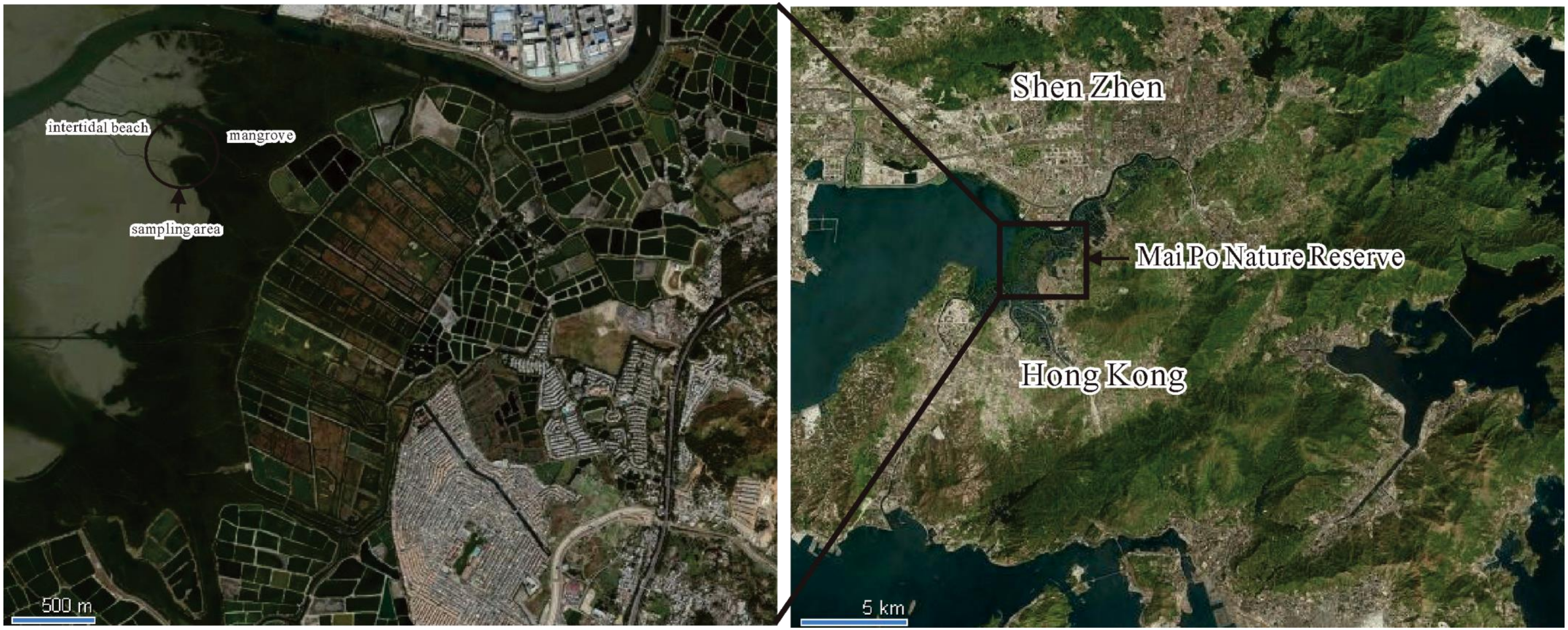

Figure S1. Geographic location and general characterization of sampling sites in Mai Po Nature Reserve located in northwestern part of Hong Kong near the borderline between Hong Kong and Shenzhen. This set of picture was acquired from Microsoft Bing Map website, generally illustrating the location and geographical patterns around Mai Po Natural Reserve (Scales bars referring to the Bing Map).

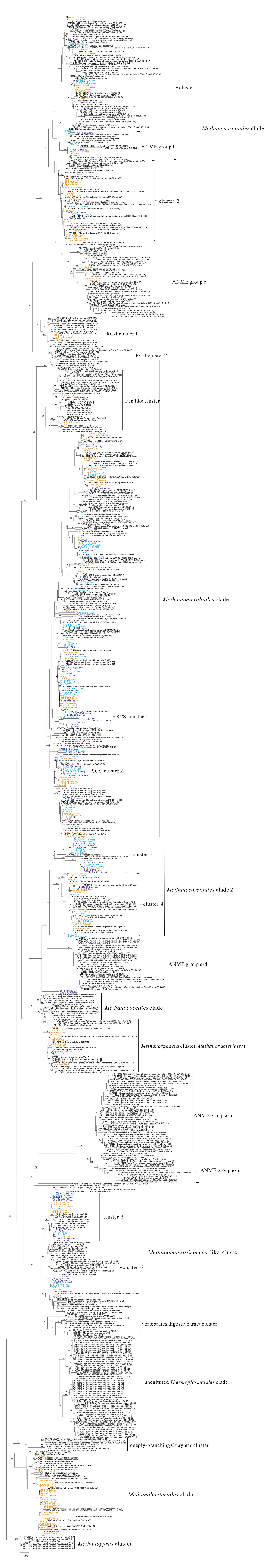
cluster 1
Methanosarcinales clade 1
ANME group f
cluster 2
ANME group e
RC-I cluster 1
RC-I cluster 2
Fen like cluster
Methanomicrobiales clade
SCS cluster 1
SCS cluster 2
cluster 3
Methanosarcinales clade 2
cluster 4
ANME group c-d
Methanococcales clade
Methanosphaera cluster(Methanobacteriales)
ANME group a-b
ANME group g-h
cluster 5
Methanomassiliicoccus like cluster
cluster 6
vertebrates digestive tract cluster
uncultured Thermoplasmatales clade
deeply-branching Guaymas cluster
Methanobacteriales clade
Methanopyrus cluster
0.05

Figure S2. Evolutionary tree based on the *mcrA* gene transcribed amino acid sequences.
The evolutionary history was inferred using the Neighbor-Joining method [1]. The optimal tree with the sum of branch length = 25.43037511 is shown. The percentage of replicate trees in which the associated taxa clustered together in the bootstrap test (1000 replicates) were shown above the branches [2]. The tree was drawn to scale, with branch lengths in the same units as those of the evolutionary distances used to infer the phylogenetic tree. The evolutionary distances were computed using the Poisson correction method [3] and were in the units of the number of amino acid substitutions per site. The analysis involved 811 amino acid sequences. All ambiguous positions were removed for each sequence pair. There were a total of 174 positions in the final dataset. Evolutionary analyses were conducted in MEGA5 [4].

1. Saitou N. and Nei M. (1987). The neighbor-joining method: A new method for reconstructing phylogenetic trees. Molecular Biology and Evolution 4:406-425.
2. Felsenstein J. (1985). Confidence limits on phylogenies: An approach using the bootstrap. Evolution 39:783-791.
3. Zuckerkandl E. and Pauling L. (1965). Evolutionary divergence and convergence in proteins. Edited in Evolving Genes and Proteins by V. Bryson and H.J. Vogel, pp. 97-166. Academic Press, New York.
4. Tamura K., Peterson D., Peterson N., Stecher G., Nei M., and Kumar S. (2011). MEGA5: Molecular Evolutionary Genetics Analysis using Maximum Likelihood, Evolutionary Distance, and Maximum Parsimony Methods. Molecular Biology and Evolution 28: 2731-2739.

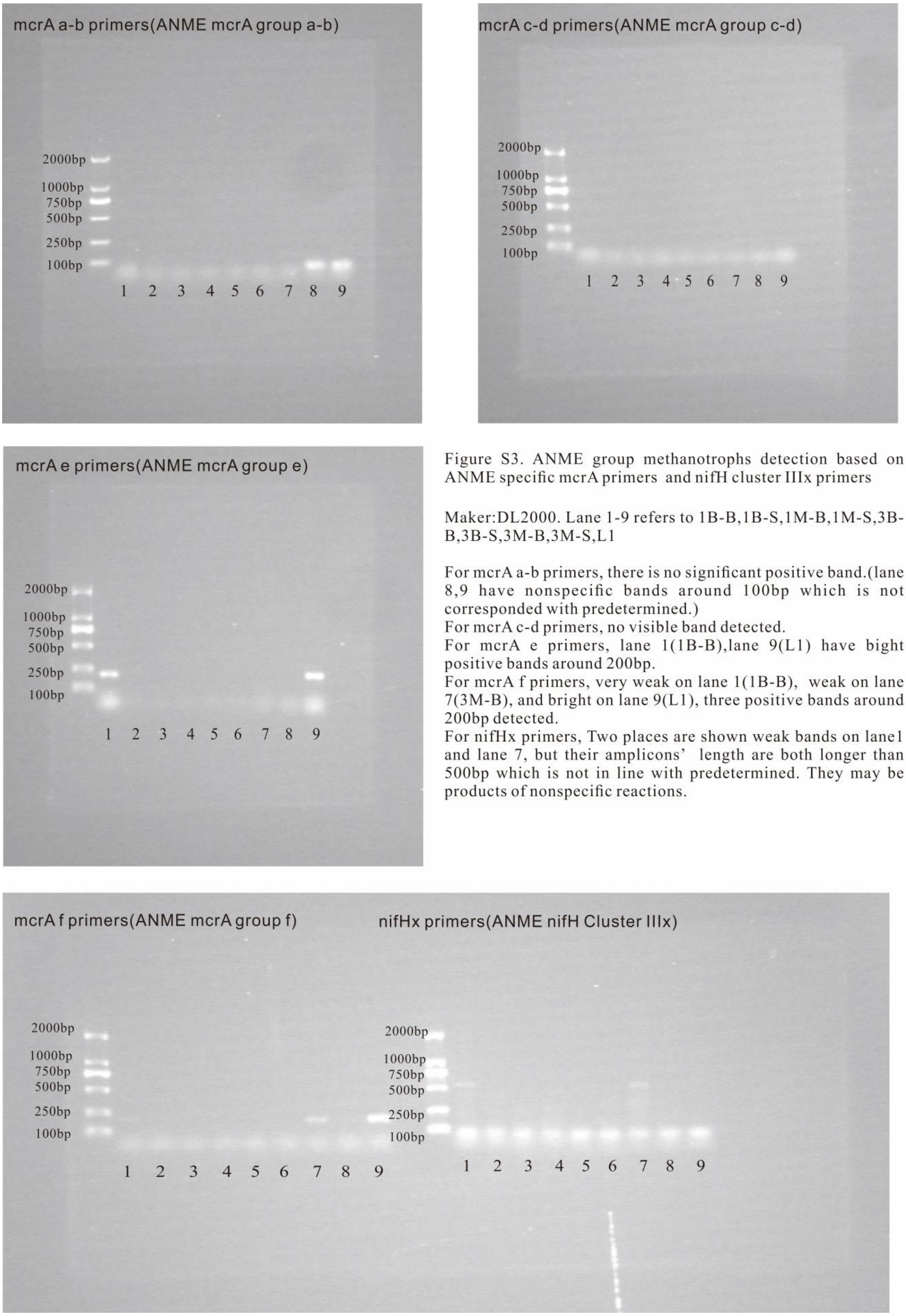

Figure S3. ANME group methanotrophs detection by semi-quantitative PCR based on ANME specific *mcrA* gene PCR primers and *nifH* gene cluster IIIx-specific PCR primers targeting samples from Mai Po Nature Reserve.

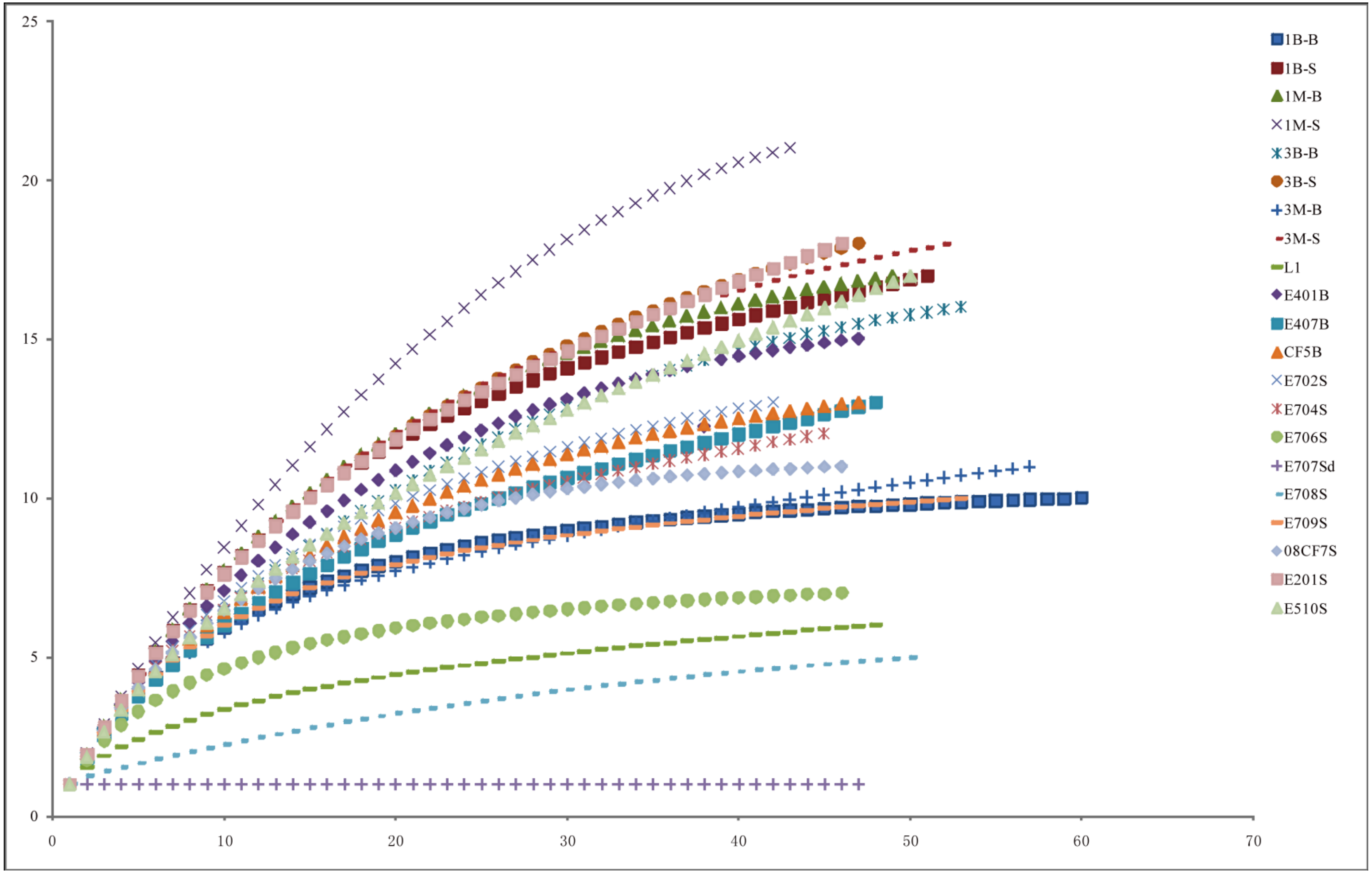

Figure S4. Rarefaction curves based on the *mcrA* gene clones retrieved from samples from nSCS and Mai Po Nature Reserve

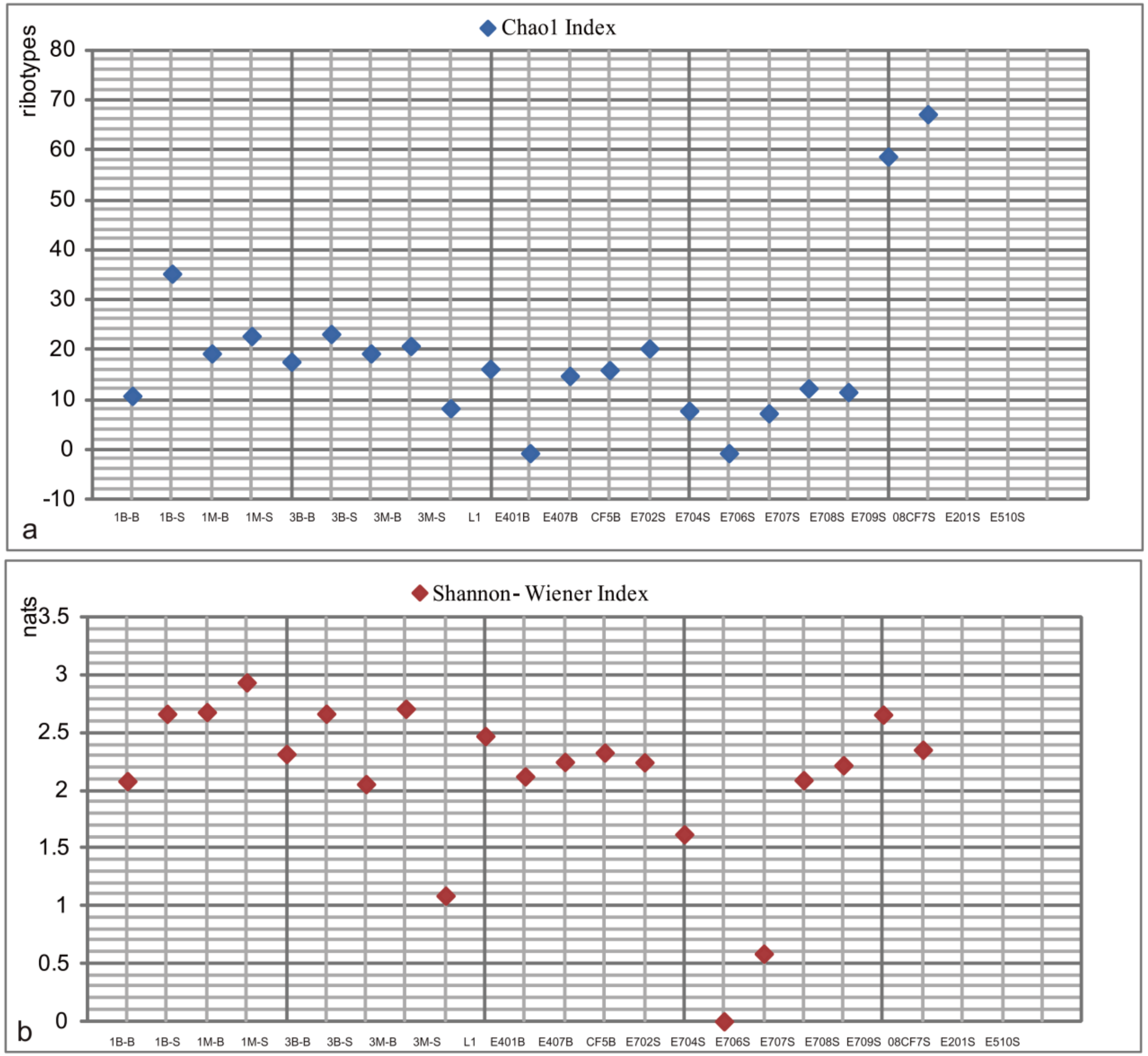

Figure S5. Chart of diversity values of based on the *mcrA* gene clones communities retrieved from each sampling sites calculated by Fastgroup II software (http://fastgroup.sdsu.edu/).

Materials and Methods

The PCR cycling condition was 95 ℃ for 2 min, following by 40 rounds of thermal cycles: 95 ℃ for 30s, 59.5 ℃ for 30s, 72 ℃ for 40s and then hold 72 ℃ for by 10 min for extension. After the first round of PCR, no visible band could be observed from the three samples. Then PCR products with the corresponding position around 500 bp were confirmed and purified. The PCR products from first round reaction were used as templates for the next round PCR in the same way. As a result, visible bands of 500 bp were observed under UV illuminator, except for sample E401B, which showed a relatively faint band. Attempted to use the product of sample E401B to conduct the downstream colony-PCR revealed no targeted fragments.

Nested PCR was used to amplify *mcrA* gene of the rest samples. The longer primer pairs (targeting around 780 bp) were chosen to amplify the sample E401B, and the remaining 8 samples from SCS by conducting 35 rounds of same PCR thermal cycles setting described above except for elongating the extension time from 40s to 50s. No visible or very faint bands were observed from the first round. Then corresponding parts at the length of 700-1000 bp regarded as expected product were collected and purified for the nested round PCR. After another round of PCR, relatively brighter bands could be observed, but they were not confirmed after PMD-18T vector ligation. A short-PCR procedure (containing pre-denaturing step of 95 ℃ for 2min, and 15 cycles of 95 ℃ 30s, 59.5 ℃ 30s, 72 ℃ 40s and then holding 72 ℃ by 10min for extension) was then introduced in order to amplify nested PCR product.
